# The mQTL hotspot on linkage group 16 for phenolic compounds in apple fruits is probably the result of a *leucoanthocyanidin reductase* gene at that locus

**DOI:** 10.1186/1756-0500-5-618

**Published:** 2012-11-02

**Authors:** Sabaz Ali Khan, Jan G Schaart, Jules Beekwilder, Andrew C Allan, Yury M Tikunov, Evert Jacobsen, Henk J Schouten

**Affiliations:** 1 Wageningen UR Plant Breeding, P.O. Box 16, Wageningen, 6700 AA, The Netherlands; 2 Wageningen University and Research Centre, Plant Research International, Business Unit Bioscience, P.O. Box 16, Wageningen, 6700 AA, The Netherlands; 3 New Zealand Institute for Plant and Food Research Limited, Mt Albert Research Centre, Auckland, 1025, New Zealand; 4 Present address: Department of Environmental Sciences, COMSAT Institute of Information Technology, Abbottabad, Postal code 22060, Pakistan

**Keywords:** Phenylpropanoid pathway, Flavonoid pathway, Transcript abundance, Apple fruits, Phenolic compounds, Leucoanthocyanidin reductase gene

## Abstract

**Background:**

Our previous study on ripe apples from a progeny of a cross between the apple cultivars ‘Prima’ and ‘Fiesta’ showed a hotspot of mQTLs for phenolic compounds at the top of LG16, both in peel and in flesh tissues. In order to find the underlying gene(s) of this mQTL hotspot, we investigated the expression profiles of structural and putative transcription factor genes of the phenylpropanoid and flavonoid pathways during different stages of fruit development in progeny genotypes.

**Results:**

Only the structural gene *leucoanthocyanidin reductase* (*MdLAR1*) showed a significant correlation between transcript abundance and content of metabolites that mapped on the mQTL hotspot. This gene is located on LG16 in the mQTL hotspot. Progeny that had inherited one or two copies of the dominant *MdLAR1* alleles (*Mm, MM*) showed a 4.4- and 11.8-fold higher expression level of *MdLAR1* respectively*,* compared to the progeny that had inherited the recessive alleles (*mm*). This higher expression was associated with a four-fold increase of procyanidin dimer II as one representative metabolite that mapped in the mQTL hotspot. Although expression level of several structural genes were correlated with expression of other structural genes and with some *MYB* and *bHLH* transcription factor genes, only expression of *MdLAR1* was correlated with metabolites that mapped at the mQTL hotspot. *MdLAR1* is the only candidate gene that can explain the mQTL for procyanidins and flavan-3-ols. However, mQTLs for other phenylpropanoids such as phenolic esters, dihydrochalcones and flavonols, that appear to map at the same locus, have so far not been considered to be dependent on LAR, as their biosynthesis does not involve LAR activity. An explanation for this phenomenon is discussed.

**Conclusions:**

Transcript abundances and genomic positions indicate that the mQTL hotspot for phenolic compounds at the top of LG16 is controlled by the *MdLAR1* gene. The dominant allele of the *MdLAR1* gene, causing increased content of metabolites that are potentially health beneficial, could be used in marker assisted selection of current apple breeding programs and for cisgenesis.

## Background

Apple (*Malus* × *domestica* Borkh) is an important source of many secondary metabolites known as phenolic compounds [1,2]. These phenolic compounds have various functions in the plant such as protection against ultra violet light [3]. The phenolic compounds such as procyanidins are polymers of flavan-3-ols. In plants they often function to prevent herbivory. They provide an astringent taste to foodstuffs and, at longer chain length, form complexes with proteins. Procyanidins are increasingly recognized for their beneficial effects on human health [4].

One of the important benefits of these compounds to consumers is their potential role against various human diseases such as cancer, coronary heart diseases, cardiovascular diseases, and diabetes [5,6].

Phenolic compounds are synthesised through the phenylpropanoid and flavonoid pathways. For procyanidins, the biosynthetic pathway largely overlaps with that of anthocyanins. These complex biochemical pathways involve a series of enzymes. Many of these enzymes, as well as the encoding genes have been functionally characterized [7-10]. The first committed step to procyanidins has been postulated to be carried out by leucocyanidin reductase (LAR) [11].

In our previous study [1] we genetically mapped phenolic compounds that were detected in peel and in flesh of ripe apple fruits. We detected a hotspot of QTLs of metabolites (mQTLs) at the top of LG16. The metabolites that mapped at this locus were procyanidins (flavan-3-ols and their polymers), and other phenolic compounds such as phenolic esters and flavonol- and dihydrochalcone derivatives. All these compounds belong to the phenylpropanoids, and one could therefore speculate that the mQTL is controlled by a biosynthetic gene from the phenylpropanoid pathway, or by a transcription factor controlling this pathway.

The aim of the present study was to unravel which gene controlled the phenylpropanoid mQTL hotspot in apple. The approach used involved an expression analysis of structural and transcription factor genes of the phenylpropanoid and flavonoid pathway. By looking closer at the draft sequence of the whole genome of the apple cultivar ‘Golden Delicious’ [12], the structural gene *leucoanthocyanidin reductase* (*MdLAR1*) and seven transcription factor genes were detected in the genetic window of the mQTL hotspot. Therefore the transcript abundances of these genes were investigated. In addition, expression profiles of the structural genes of the phenylpropanoid and flavonoid pathways outside of the mQTL hotspot were studied. A strong positive correlation between the expression level of the *MdLAR1* gene and the level of metabolites that mapped at LG16 was observed. This was not found for any of the other genes studied. This indicates that the *MdLAR1* gene is the major candidate gene controlling the mQTL hotspot on LG16. Further evidence is provided by the fact that the *MdLAR1* gene is the only structural gene of the phenylpropanoid and flavonoid pathways that resides in the mQTL hotspot.

## Methods

In this study, fruits from the segregating F1 population derived from the cross between the cultivars ‘Prima’ and ‘Fiesta’ were used. This population was used in our previous study too, in which the mQTL hotspot and other mQTLs were detected [1].

### Selection of genotypes and harvesting of fruits for gene expression studies

**Figure 1:**
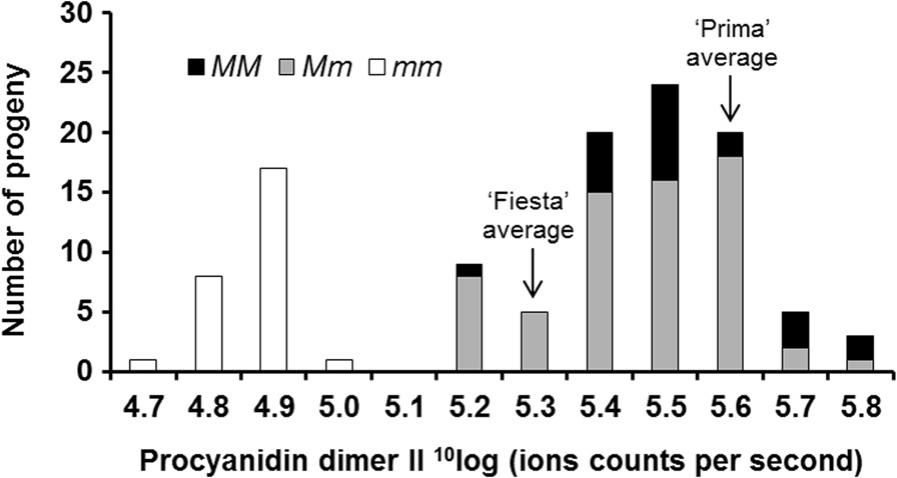
**The procyanidin dimer II contents (ion counts per second) of the ripe fruits of the genotypes that were used for studying gene expression during fruit development[**[1]**].** This metabolite segregated clearly in the F1 progeny in both peel and flesh, and represents the metabolites that mapped in the mQTL hotspot on LG16. The genotype classes are *mm* (homozygous recessive), *Mm* (heterozygous dominant) and *MM* (homozygous dominant).

**Figure 2:**
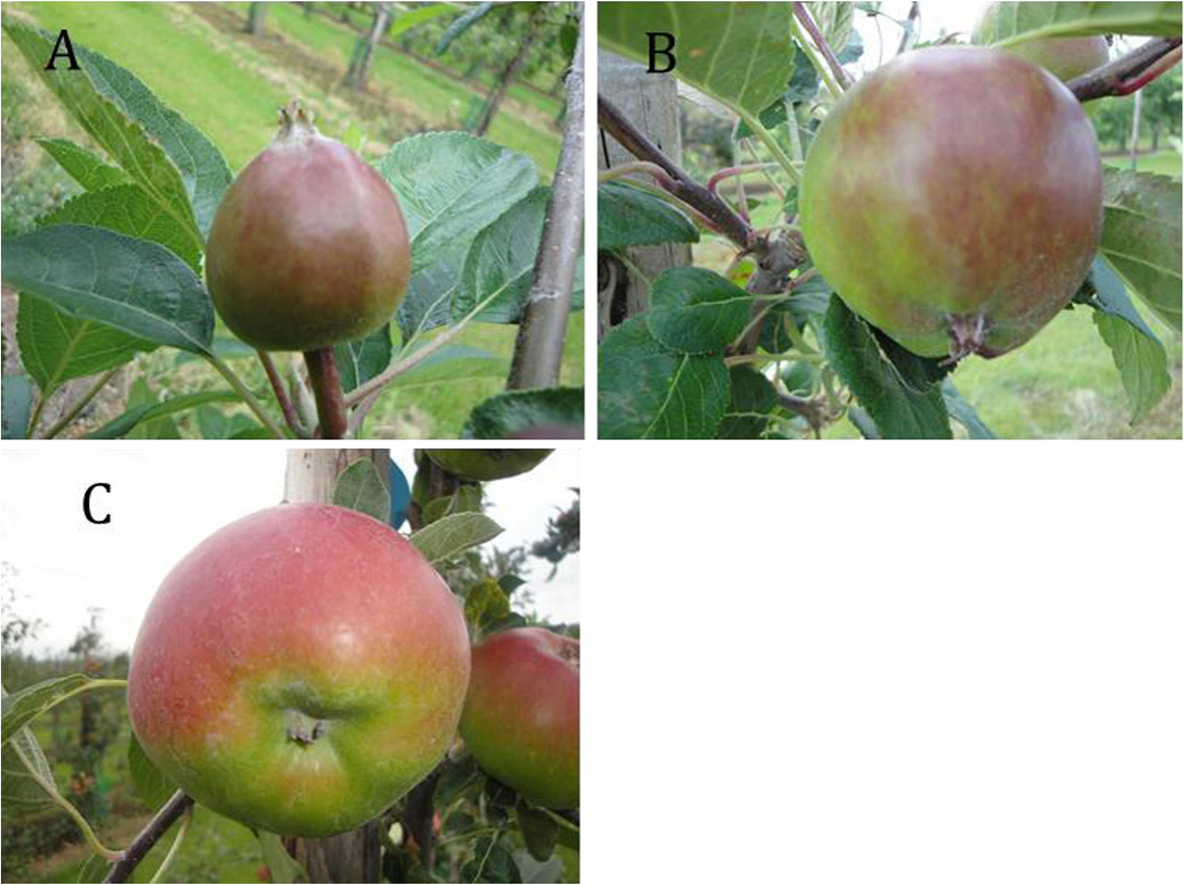
**The three developmental stages of growth of apple fruit used for gene expression in this study.A**= 34 Days After Full Bloom **(**DAFB), **B**= 60 DAFB and **C**= 95 DAFB.

We selected genotypes based on the clear genetic segregation of metabolite ‘procyanidin dimer II’ (Additional file 1). This metabolite belongs to the phenylpropanoid pathway and its concentration showed a clear segregation (Figure 1). It was mapped at the mQTL hotspot on LG16 as described by Khan et al. [1]. This metabolite was used as representative metabolite for all other metabolites that mapped at the mQTL hotspot. The progeny genotypes from the cross ‘Prima’ × ‘Fiesta’ were divided into two groups based on ‘procyanidin dimer II’ clear segregation, *i.e.* one group (Group A) having low content and another group (Group B) having high content of ‘procyanidin dimer II’ (Additional file 2). The trees were at full bloom from 26-30^th^ April, 2010. Fruits from trees in the trial orchard located in Randwijk, the Netherlands were harvested at three developmental stages (Figure 2), eight fruits per tree for each developmental stage, and subsequently peeled off and were stored as described in our previous article [1]. Ten genotypes were selected from ‘Group A’ and nine genotypes were selected from ‘Group B’ with two trees per genotype (two biological replicates; Additional file 1). As references, the parents of the segregating F1 population were included in the analysis with two trees for each parent. Both parents belong to the heterozygous class of genotypes. The genotype numbers and genotype classes used in this study are given in Additional file 1. The sizes (diameter) of individual fruits were measured at each developmental stage using an electronic digital caliper, model VWRI819-0012 of Control Company. The average size at 34 Days After Full Bloom (DAFB) was 24 mm, at 60 DAFB 40 mm, and at 95 DAFB 62 mm (Figure 2; Additional file 1).

There were three classes of genotypes based on co-segregating genetic markers: *MM* was the homozygous dominant class. These progeny inherited from each parent one dominant allele for increased content of the procyanidin dimer II. *Mm* was the heterozygous class, which has one dominant allele from one parent and one recessive allele from the other parent. The heterozygous progeny had high content of the metabolite too. The third class is the homozygous recessive class *mm*. This class received both recessive alleles from the two parents, and showed a low content of the metabolite.

### RNA isolation from apple fruits

Total RNA was isolated from peel and flesh of apple fruits separately according to the CTAB method described by Asif et al. [13]. The RNA quantity was measured on NanoDrop® spectrophotometer model ND-1000 from isogen lifescience scientific company as explained by Khan et al. [14] and the RNA quality and quantity were measured by running 2 μl of the RNA sample on a 1.5% agarose gel. First single-strand complementary DNA (cDNA) was synthesized using iScript™ cDNA Synthesis Kit (Bio-Rad) according to the manufacturer’s manual.

### Selection of genes for qRT-PCR studies and primer design

The *MdLAR1* gene was detected in the middle of the genetic window for the mQTL hotspot at the top of LG16 [1]. Therefore this structural gene was included in the gene expression study*.* In addition, the other structural genes of the phenylpropanoid and flavonoid pathways were included (Table 1). Further more, all putative transcription factor genes that were located within the genetic window of the mQTL hotspot were included. Also we added putative transcription factor genes which neighboured this genetic window using the ‘Golden Delicious’ genome sequence [12], or which showed high homology to genes that are known to regulate the phenylpropanoid and flavonoid pathways in other plant species (Table 1). Primer pairs for structural genes of the phenylpropanoid and flavonoid pathways were kindly provided by Plant and Food Research, New Zealand. The primer pairs for the other candidate genes were designed with the online available program ‘Primer3Plus’ (http://www.bioinformatics.nl/cgi-bin/primer3plus/primer3plus.cgi). The primer names and their forward and reverse sequences are given in Table 1. The primers were tested using q RT-PCR in the same way as explained by Khan et al. [14]. The qRT-PCR products were checked for quality by checking their clear single peak in the melting curve and a clear band of the expected amplicon size on 1.5% agarose gel.

**Table 1:** Genes that were included in the expression analysis

**Group**	**Gene name**	**Gene ID**	**Forward primer (5’→ 3’)**	**Reverse primer (5’→ 3’)**	**LG on “Golden Delicious”**	**Gene position on LG (kbp)**
Structural genes	*MdLAR1pair1*	MDP0000171928	GTGGTTAACGGAGGCACAGT	CCGAGGAGAAAGGACTACCC	LG16	1536
	*MdLAR1pair2*	AY830131	GTGCTTCGATGGCTTTCTTC	TAACAAGCTCACCCCCAAAC	LG16	1530
	*MdLAR2*	AY830132	ATGCCACAATCGTGTCAAAA	GGCTGGCTTCAGCTACAAAC	LG13	2860
	*MdPAL*	ES790093	CGAGGAGTGTGACAAGGTGTTCCA	AGGAATGCAGCATGTAAACCGTGAC	LG4	8075
	*MdC4H*	EB135197	GGACGTTTAGTCCAGAACTTCGAGCT	ACTTCATCACAATGGTGGAATGCTTC	LG11	4614
	*Md4CL*	EB122629	CATAAACAGTGTCCCCAAGTCAGCAT	AGTGTTCCTACAAGCCTTCCCGATAA	LG11	5126
	*MdCHS*	CN944824	GGAGACAACTGGAGAAGGACTGGAA	CGACATTGATACTGGTGTCTTCA	LG9	15948
	*MdCHI*	CN946541	GGGATAACCTCGCGGCCAAA	GCATCCATGCCGGAAGCTACAA	LG1	16132
	*MdF3H*	CN491664	TGGAAGCTTGTGAGGACTGGGGT	CTCCTCCGATGGCAAATCAAAGA	LG5	20985
	*MdDFR*	AF117268	GATAGGGTTTGAGTTCAAGTA	TCTCCTCAGCAGCCTCAGTTTTCT	LG12	21890
	*MdANS*	AF117269	GATGAAGGGAGGCTGGAGAAAG	GTGGAGGATGAAGGTGAGTGC	LG6	13776
	*MdFLS*	EB137300	TCAGATGGAGATAATGAGCAATGGAAA	ATTAACGGGGTTCACAAGCTGTGG	LG8	15333
	*MdANR*	EB125405	TCGCTGGCTTATGATCCTCCTGTT	CCGTTTGCCAAACTCAGCAAATTA	LG5	2243
	*MdHCTchr9*	MDP0000851389	CGATGCTGTTTTCAGAACCA	GCAGCAGACGAGGATGATTA	LG9	24590
	*MdC3Hchr8*	MDP0000466557	CAAAGGAGGTGCTCAAGGAG	TGGACTCGACCATAGCAGTG	LG8	29024
	*MdF3’Hchr6*	MDP0000539956	ACTCTCTTCATGCGCTTGGT	TGCCTATCCTCACCCAAAAG	LG6	22805
	*MdF3’Hchr14*	MDP0000370951	ACCATTAAACCCCAACAACG	ATCACGGTTTGGAGCTCTTG	LG14	27562
	*MdUFGT*	AF117267	AAGGTCTCTCCAATGTACGAAT	AGGAGTTTGTTGACTTTGGACT	LG1, LG7	29053, 26292
TF genes at mQTL hotspot	*MdMYB1361*	MDP0000375685	CTGGGGGTTCAAGTAGTCCA	CTCCGTGGTGGCTTGATAAT	LG16	1361
	*Mdb-HLH1967*	MDP0000261293	GATACGGCATCATTCCTGCT	GCCTGAGGATTTCCAACAAA	LG16	1967
	*Mdb-HLH1881*	MDP0000154272	CTCAACCGGGACTTATCCAA	GCTCATCCTCCCACACATTT	LG16	1881
	*Mdb-HLH1543*	MDP0000319726	GAGCTGAAACGCCAAACTTC	CGGTGATGAACAACACGTTC	LG16	1543
	*MdAP21480*	MDP0000939633	GCACCTTCAACGAAGAGGAC	GACTTGGAGTGGGAGCTCAG	LG16	1475
	*MdG2L61440*	MDP0000202657	AGACCGACTCCAACAATTCG	GGACTGGTGGTGAGACCTGT	LG13	2702
	*MdbZIP1380*	MDP0000250967	CTGTTTCTGGCAAAGGCTTC	CCATCAACATTGCAGTGGAC	LG16	1376
Transcription factor genes outside the mQTL hotspot	*MdCOL1220*	MDP0000185616	TGATTTTATGGGGTGCCAAT	TAATCACCGCCTCGTAATCC	LG16	1224
	*Mdb-HLH1080*	MDP0000725991	GGCCAATGACACCTCCTTTA	TGAGCTGTGGAATGAGCAAC	LG16	1084
	*MdMYB1070*	MDP0000659260	ACTCCGCAAGAACAGCTCAT	GCTGTTCGACTCGATGTTCA	LG16	1058
	*MdC2H21020*	MDP0000183099	CCTCCTCACCTCCTCTCTCC	CCCGGCTCTGTTGTAGTACC	LG16	1021
	*MdC2H21000*	MDP0000283750	ATTCAGCAAGTTGGGTGTCC	TTTGCTTTGTGCAGTTGAGG	LG16	1003
	*MdMyb5a.A*	MDP0000791870	GGGGAGGAGGAAATGAAGAG	CAGAGTCCCAGCCAAATGTT	LG3	967
	*Mdb-HLH33*	MDP0000309179	GGAGACATCAAAACCCGAAA	TGAAGGACATGCAAAGCAAG	LG15	37144
	*MdTTG1*	MDP0000906307	GACCCGGATACCCTTTCAAT	AAACTCGCTGGTCTTGCTGT	LG1	28763
	*Mdb-HLH3*	MDP0000225680	GTCGCCATTGGTAAGGCTAA	CCACCGTGGTCTCAATTCTT	LG11	32877
	*MdMyb9*	MDP0000210851	GGCCACTAGGTTGACCAAAA	ATCATCGCAGCCAAAGTTCT	LG8	9430
	*MdMyb11.A*	MDP0000437717	TGAAGGTCGCTCATGTTCTG	ATTCACCGCCTGGTTTAGTG	LG13	30373
	*MdGAPDH*	CN494000	GCTGCCAAGGCTGTTGGAA	ACAGTCAGGTCAACAACGGAAAC	LG16, LG13	5636, 7648

17 putative structural genes of the phenylpropanoid pathway, seven putative transcription factor genes in the genetic window of the mQTL hotspot on LG16, and 11 putative transcription factor genes outside this mQTL hotspot were studied. *MdGAPDH* was used as a reference gene. The last four digits in some transcription factor genes represent their positions on the genome in kbp.

In view of the importance of *MdLAR1,* two primer pairs were designed for this gene in two different non-overlapping regions. By means of these primer pairs, the two fragments were amplified and sequenced for each genotype class. This was done for verification of the gene specificity of the primers, since *MdLAR1* on LG16 and *MdLAR2* on LG13 (Table 1) show 62% similarity at the nucleotide level. The sequences of *MdLAR1* and *MdLAR2* in ‘Prima’ × ‘Fiesta’ showed good alignment with sequences from cv. ‘Golden Delicious’ on which the primers for qRT-PCR were designed.

### Performing q RT-PCR and data analysis

Gene expression was measured using Fluidigm Dynamic Array integrated fluidic circuits for cDNA samples from peel and flesh for the genotypes and development stages mentioned in Additional file 1. Fluidigm used the BioMark™ System and Evagreen DNA binding dye (http://www.fluidigm.com). Three 96×96 Dynamic Arrays of Integrated Fluidic Circuits, comprising 48 primer pairs in two replicates were used. The q RT-PCR set up for the reference gene and other control samples, and data analysis was performed as described by Khan et al. [14].

### Correlation network analysis

The correlation coefficients were calculated between the contents of seven metabolites representative for different branches of the phenylpropanoid and flavonoid pathway, and for the expression of 18 structural genes and 18 transcription factors possibly involved in these pathways. Before calculation of the correlation coefficients, the data were ^10^log transformed for normalisation purposes. Scatter plots were made between the different ^10^log transformed variables, in order to make sure that outliers did not bias correlation values, and to check the distributions.

Visualization of the correlation network was performed by the Pajek software package (http://pajek.imfm.si/doku.php). Besides a biological quantitative pattern which is observed in a set of samples as the result of physiological processes, data may have a particular embedded ‘experimental pattern’ which is due to the experiment performance, such as extraction errors and measurement or calibration errors. So, different analytical methods run on the same set of samples may give different experimental patterns. Therefore, correlations between variables observed within particular experiments may be stronger than correlations between variables from different experiments. Here we have a correlation matrix of two different experiments and, therefore, three types of correlations (sub-matrices) are present: gene-to-gene correlations, metabolite-to-metabolite correlations and gene-to-metabolite correlations. Lower correlation coefficients might be expected in the third sub-matrix due to interference of different experimental patterns. To compensate for this effect and to obtain a balanced correlation network we standardized correlation coefficients separately for each of the three sub-matrices. For these a maximum positive and negative correlation coefficients *r* were found in each sub-matrix and then were set to 1.0 and −1.0, respectively. Other correlation coefficients of each sub-matrix were expressed relative to their maximum ones. The standardized correlation coefficients are further denoted as *r*_s_.

## Results

### Association between expression of structural genes of the phenylpropanoid/flavonoid pathways and concentrations of metabolites that mapped at the mQTL hotspot

**Figure 3:**
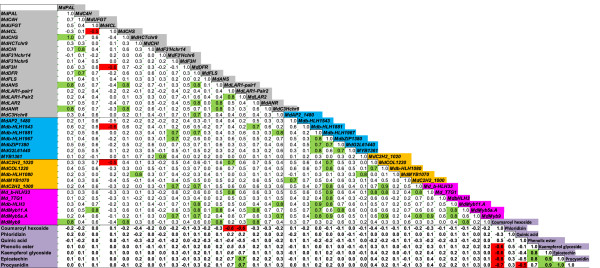
**Correlations between the expression levels of genes and metabolite contents in peel.** The different groups of genes have different colors. Grey: structural genes of the phenylpropanoid pathway. Blue: the putative transcription factor genes that were detected in the mQTL hotspot; Yellow: the putative transcription factor genes that were outside the mQTL hotspot but on LG16; Pink: putative transcription factor genes of the phenylpropanoid pathway that are not located on LG16 but are at other regions of the genome. The metabolites are highlighted in purple and their correlations with the expression levels of all the genes are given in bold text. The metabolites contents (ions count per second) and expression data (expression relatively to the reference gene *MdGAPDH*) were ^10^log transformed before calculation of the correlation coefficients. Scatter plots (data not shown) were made to evaluate the distributions and to exclude outliers that might strongly affect the correlations. The correlations between the expression level of *MdLAR1* and metabolite content are given in bold italics text. Green color shows other correlations (*r* > 0.7). Red color highlights negative correlations between **−**0.5 and **−**1.0. For each metabolite group that had an mQTL at the LG16 hotspot, a representative metabolite is included in this correlation matrix. Similar results were also found in flesh (Additional file 3).

None of the 17 structural genes of the phenylpropanoid and flavonoid pathways which were evaluated for gene expression, showed a significant correlation with the content of procyanidin dimer II, except for the *MdLAR1* gene (Figure 3). This was observed both in peel and flesh tissues and at the three different fruit developmental stages (Additional file 3). For *MdLAR1* we evaluated the expression using two different primer pairs, annealing at different places in the *MdLAR1* gene. For both primer pairs, the measured expression showed a positive correlation with procyanidin and other phenolic metabolites that mapped in the mQTL hotspot (Figure 3). However, the metabolites quinic acid and coumaroyl hexoside appeared to have a negative correlation with the *MdLAR1* expression (Figure 3).

The progeny that had inherited the recessive alleles for low procyanidin dimer II content (*mm*), showed a low expression of *MdLAR1* throughout fruit development, both in peel and flesh (Figure 2). However, the heterozygous group (*Mm*) showed a higher expression, compared to the homozygous recessive (*mm*) group, whereas the homozygous dominant progeny (*MM*) with high content of procyanidin dimer II showed the highest expression of *MdLAR1* (Figure 2). The expression level of *MdLAR1* was highly significantly, positively correlated with procyanidin dimer II content, according to Student’s t-test (*P <* 0.1%). On the average, the *MM* genotypes had a four times higher content of this metabolite at the ripe stage compared to the *mm* genotypes (Figure 1), both in peel and flesh.

No significant correlation was detected between transcript abundance of the other evaluated genes at the one hand with the concentration of procyanidin dimer II at the other hand (Figure 3).

The transcript abundance of *MdLAR1* was also significantly correlated with the other metabolites that mapped at the LG16 hotspot (Figure 3). However, the other studied genes did not show this high correlation with any of the metabolites at the mQTL hotspot. This suggests that the *MdLAR1* gene is the gene controlling the mQTL of procyanidin dimer II, and of all other phenolic compounds that mapped at this hotspot on LG16.

### Association between expression of transcription factor genes and concentrations of metabolites that mapped at the mQTL hotspot

The finding that compounds from different locations in the pathway mapped at the same mQTL hotspot [1] could suggest that a transcription factor was involved in the mQTL. At the mQTL locus, seven candidate transcription factor genes were identified (Table 1). However, there was no clear correlation for any of these candidate transcription factor genes with the procyanidin dimer II content in peel and flesh (Figure 3). This indicates that the evaluated transcription factor genes at the mQTL hotspot were not responsible for this hotspot.

In addition, 11 more candidate transcription factor genes were identified throughout the genome, or on homology to known transcription factors involved in the phenylpropanoid and flavonoid pathway (Table 1). No clear correlation was found between the expression of any of these putative transcription factor genes and the metabolites that mapped at the hotspot (Figure 3). This indicates that transcription factor genes outside the mQTL hotspot were not controlling this hotspot either.

### Associations between expression of structural genes and transcription factor genes

The correlation matrix (Figure 3) shows that the expression levels of many genes were correlated to one another. As an example, the expression of the structural genes *MdPAL, MdC4H, MdUFGT, MdCHS, MdCHI, MdF3H, MdDFR, MdLAR2* and *MdANS* were positively correlated to one another. This cluster of structural genes showed also a positive correlation with the expression of the three transcription factor genes *b-HLH1543, MdMyb11.A* and *MdMyb9.* This suggests that these three transcription factor genes may regulate this cluster of structural genes, but did not control the mQTL hotspot on LG16.

**Figure 4:**
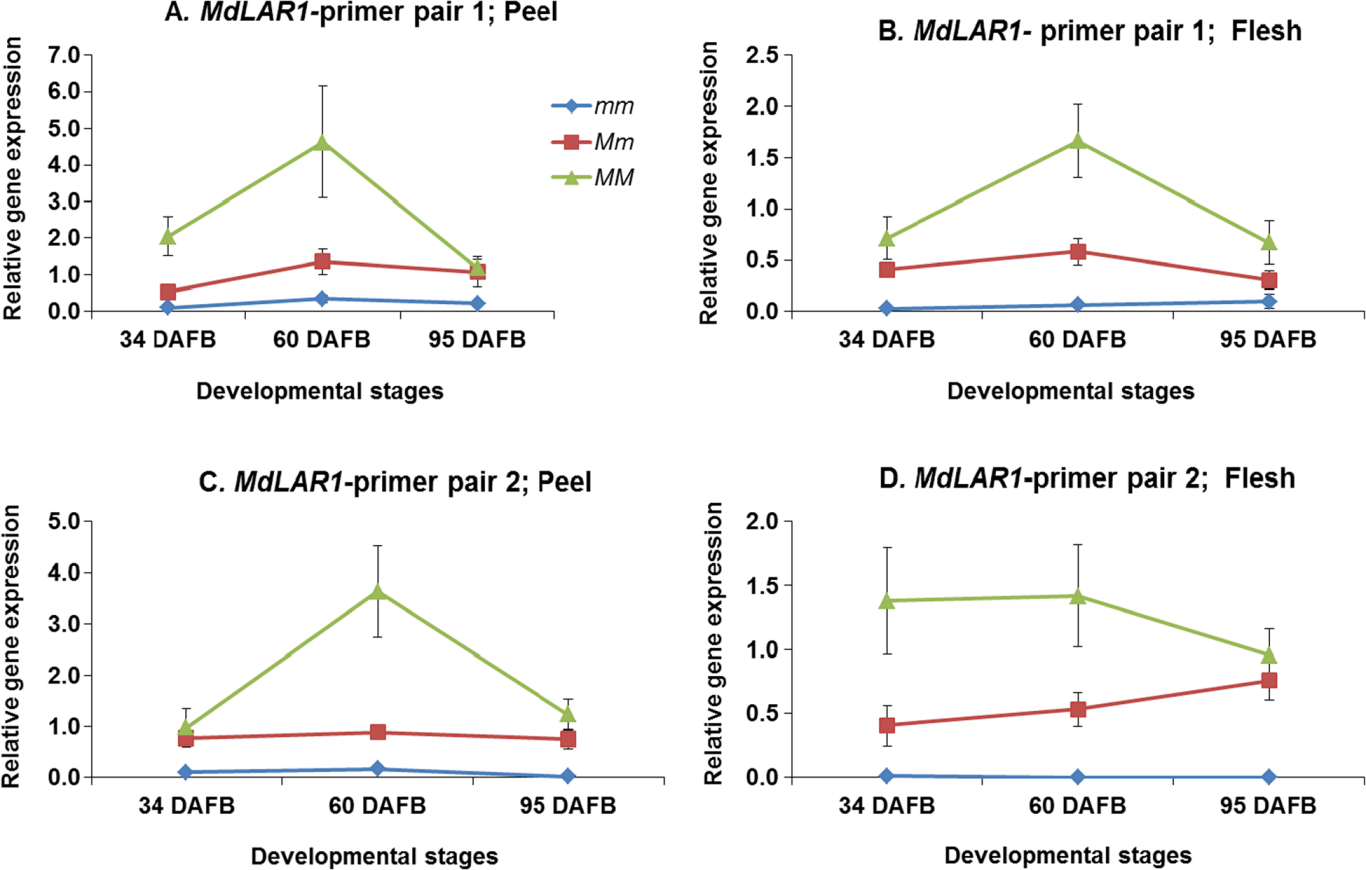
**Relative gene expression of the structural gene MdLAR1.** Figures **A** and **B** are the results with primer pair 1 for this gene in peel and flesh and Figures C and D are the results for primer pair 2 in peel and flesh respectively. The three different developmental stages are given on the horizontal axis as 34, 60 and 95 DAFB.

**Figure 5:**
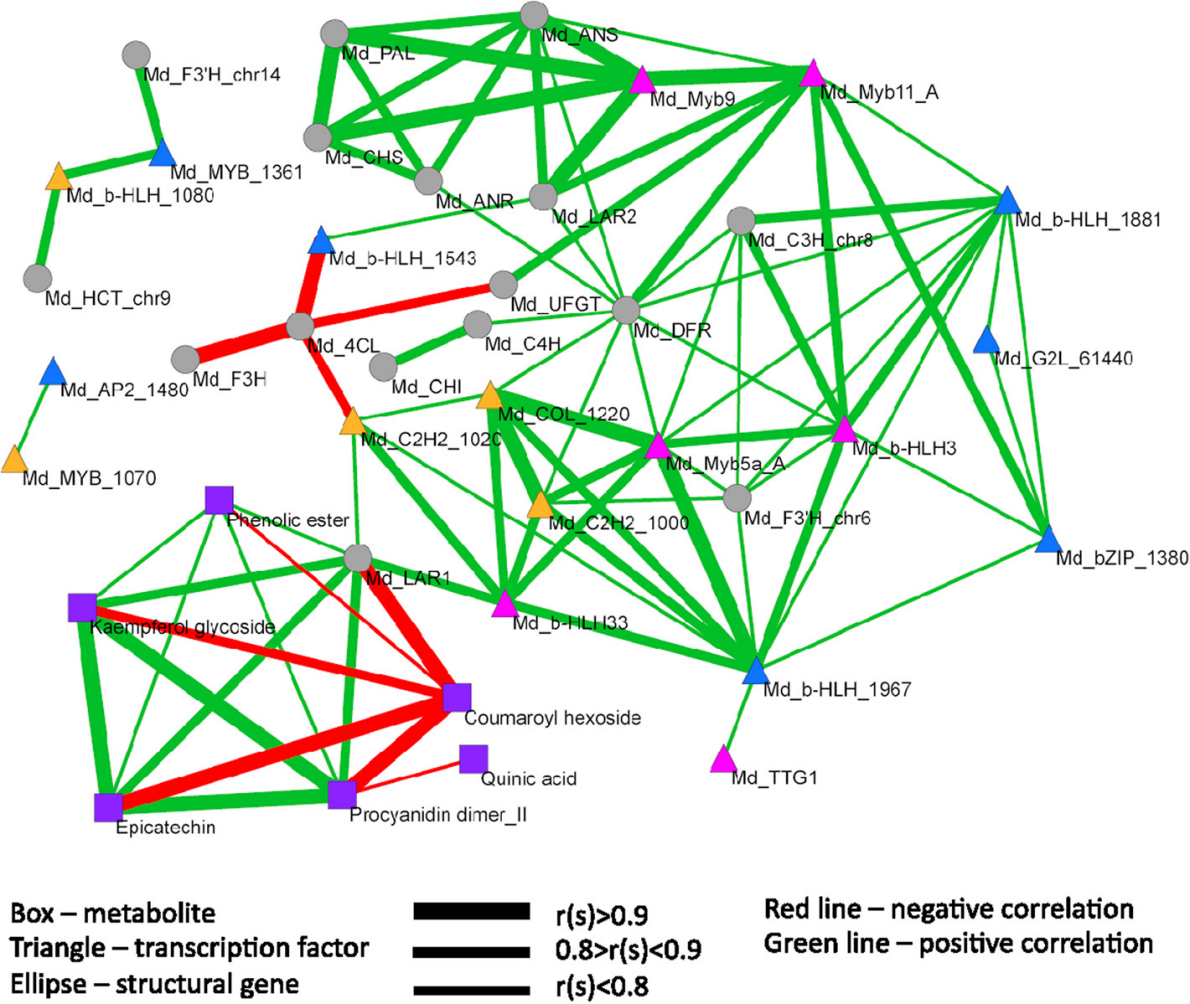
**A correlation network of 7 metabolites and 36 candidate genes of the phenylpropanoid and flavonoid pathways in apple peel.** Different figures represent metabolites – purple box; structural genes – grey ellipse; transcription factors inside the mQTL hotspot on LG16 – blue triangle; transcription factors on LG16, but outside the mQTL hotspot – yellow triangle; transcription factors elsewhere in the apple genome – pink triangle. Red connection lines represent negative correlations and green lines – positive correlations and thickness of the lines correspond to the standardized correlation coefficient rs.

We visualized the correlations of Figure 3 in a network (Figure 4). This network is divided into two clusters. The first cluster shows the correlations between the metabolites that mapped at the mQTL hotspot of LG16. The green lines in this cluster show positive correlations, and the red lines negative correlations. These colours resemble the mapping results, depicted in Figure 5. Striking in this cluster of metabolites is the presence of one gene only, i.e. *MdLAR1*. This gene is located in the centre of the mQTL hotspot of these metabolites.

In the second cluster of the network, many structural genes and transcription factors appear to be connected to one another (Figure 4). Several genes in the network are important nodes, and are connected to many other genes. This is especially the case for *MYB* transcription factors, such as *MdMYB9, MdMYB11_A,* and *MdMYB5a_A,* and for *b*-*HLH* transcription factors, such as *b-HLH1881, b-HLH1967,* and *MdbHLH33* (Figure 4). Probably these transcription factors regulate many structural genes in the phenylpropanoid pathway. However, none of these transcription factor genes is directly connected to metabolites in the first cluster. In spite of the important regulatory roles of the mentioned *MYB* and *b-HLH* transcription factor genes in the phenylpropanoid and flavonoid pathway, they were not responsible for the mQTL hotspot.

## Discussion

### Aim of the study

In our previous study [1] we mapped phenolic compounds in ripe fruits of a segregating F1 population derived from the cross between cultivars ‘Prima’ and ‘Fiesta’. There appeared to be a strong hotspot of mQTLs at the top of LG16. Annotation of the metabolites showed that the compounds that mapped on the LG16 hotspot belong to the phenylpropanoid and flavonoid pathways (Figure 5).

We wanted to discover which gene(s) controlled this mQTL hotspot. Therefore, in the present research, transcript abundances for the candidate genes in the mQTL region were measured in progeny genotypes that segregated for these mQTLs. In addition, structural genes of the phenylpropanoid and flavonoid pathways and putative transcription factor genes that are candidates for regulating these pathways and located elsewhere were evaluated as mentioned in the Methods section in detail.

### *MdLAR1* seems to be the only gene that can explain the mQTL hotspot on LG16

**Figure 6:**
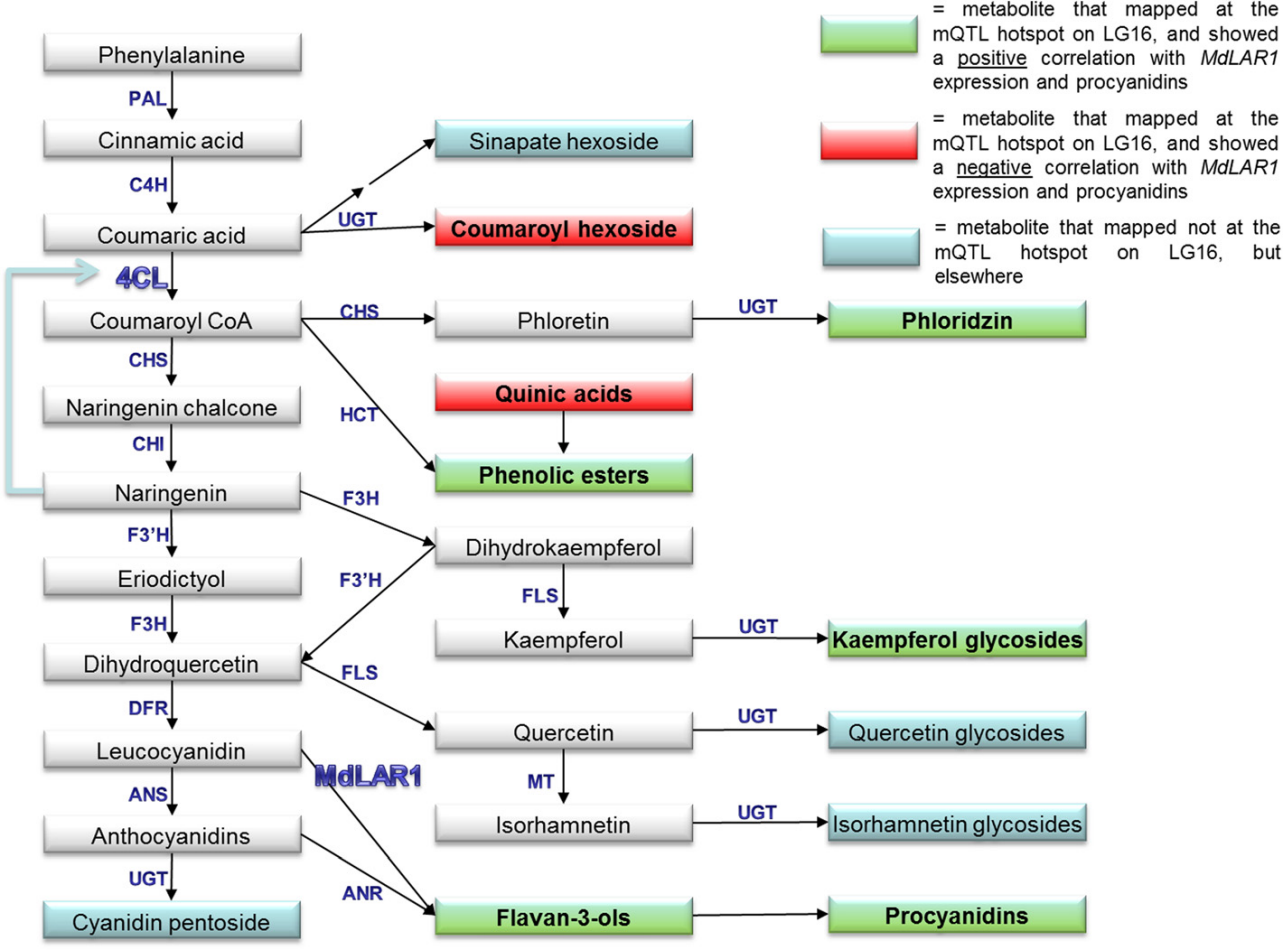
**Simplified scheme of the mapping results of phenolic compounds in mature apple fruits according to Khan et al.**[1].

As shown in Figure 3, *MdLAR1* was the only gene for which the expression was clearly correlated with the metabolite content, both in peel and flesh. None of the other genes showed a clear correlation with procyanidin dimer II content. Moreover, Figure 6 shows clearly that the expression of *MdLAR1* was low for the genotypes that had inherited the recessive alleles (*mm*), and had low content of the representative metabolite procyanidin dimer II. The progeny that had inherited one or two dominant alleles (*Mm, MM*) had higher expression levels of *MdLAR1* and higher content of procyanidin dimer II (Figure 6). This pattern was observed both in peel and in flesh. This was not the case for any of the other genes studied, which suggests that *MdLAR1* was responsible for the hotspot of mQTLs on LG16. Furthermore, it indicates that *MdLAR1* exerted its influence by means of its expression level. Recent findings in grape also showed a genetic association between a *LAR* gene and a procyanidin QTL [15].

The procyanidin content was higher in the flesh compared to the peel (Figure 1). However, the expression of *MdLAR1* was lower in the flesh compared to the peel (Figure 6). A possible explanation is the fact that flavonols and anthocyanins are produced in the peel only. These may compete for the pool of available substrates, leading to relatively lower procyanidins level.

### How can *MdLAR1* explain the observed mQTLs?

The *MdLAR1* gene clearly explains the mQTL for procyanidin content, as LAR from leguminosal species has been implicated in the synthesis of catechin, a building block for procyanidins [11]. Remarkably, we found several mQTLs in the same hotspot on LG16 for metabolites (kaempferol glycosides, phloridzin, phenolic esters) that are synthesized by different branches from the phenylpropanoid pathway [1] (Figure 5). Since *LAR* is not known to be involved in the biosynthesis of these other metabolites, the observed differential *LAR* expression does not provide a straightforward explanation for the presence of the mQTLs of these more upstream metabolites.

One could speculate about the effect that *LAR* overexpression may have effect on the total flux through the phenylpropanoid pathway. We note that the positively associated mQTLs (procyanidins, dihydrochalcones, phenolic esters and kaempferol glycosides) all map downstream of coumaroyl-CoA ligase (4CL) in the pathway (Figure 5). A metabolite that maps upstream of 4CL is coumaroyl hexoside, for which the level was negatively correlated with e.g. procyanidins. This appears also from Figure 3.

In apple, no *4CL*-like gene is located at the mQTL hotspot [12]. Moreover, the expression of the tested *4CL* gene did not correlate with the metabolites that mapped at the hotspot. One explanation may be that *MdLAR1* overexpression relieves a feedback mechanism on the enzymatic activity of 4CL. 4CL is known to be feedback inhibited by metabolites from the phenylpropanoid pathway, such as naringenin [16]. Possi bly, the enhanced MdLAR1 activity will lead to depletion of pathway intermediates such as naringenin, which may thus activate 4CL activity and lead to a higher general flux, from coumaroyl glycoside towards the downstream metabolites. The support for such a mechanism needs extensive experimentation, which is outside the scope of this article.

An unlikely, but still possible alternative explanation for the mQTL hotspot could be that a transcription factor at the mQTL hotspot regulated the expression of *MdLAR1.* As we did not see any differencial expression of the transcription factor genes at the mQTL hotspot, the different alleles of that transcription factor gene would not differ in expression levels, but theoretically could differ in effect of the protein. Further, that transcription factor might have influenced *4CL* paralogous that were not covered by the used primer pair. We do not regard this as a likely explanation, but it cannot be completely excluded.

### Transcript abundances of several structural genes and transcription factor genes were correlated

*MdANR* also contributes to the synthesis of procyanidins (Figure 5). The expression level of this gene significantly correlated with expression of several structural genes such as *PAL*, *CHS*, *DFR*, and *ANS* (Figures 3 and 4). Moreover, there was a clear correlation between the expression of these structural genes, and the expression of the transcription factor genes *MYB9* and *MYB11* (Figures 3 and 4). Possibly, these transcription factors regulated the mentioned structural genes. However, the transcript abundances of none of these structural or transcription factor genes did correlate significantly with the metabolite abundances that mapped at the mQTL hotspot on LG16 (Figure 3). This indicates that these structural genes were not the bottleneck for the pathway, whereas probably *MdLAR1* was the limiting factor in the progeny that had inherited both lowly expressed alleles of this gene (*mm*). Presumably, the bottleneck was (partly) removed in case of presence of one or two higher expressed alleles of *MdLAR1* (*MM, Mm*).

### Applications

The dominant allele of the *MdLAR1* gene, causing increased content of metabolites that are potentially health beneficial, could be used in marker assisted selection of current apple breeding programs. This selection could be made at seedling stage. This would reduce the production costs for the breeders by discarding the undesired seedlings at earlier stage of growth, whereas in classical breeding only after six years, when trees start to bear fruits, selection on fruit content is possible. Another possibility is to clone the dominant allele or alleles for engineering increased content of metabolite(s) into existing apple cultivars by different transformation technologies including cisgenesis [17,18].

## Conclusions

Our results indicate that *MdLAR1* is the most likely candidate gene responsible for the mQTL hotspot for phenolic compounds on LG16 of apple, both in peel and flesh. Increased levels of metabolites downstream of *MdLAR1*, such as the flavan-3-ols epicatechin and procyanidin dimer II may be directly caused by increased transcript abundance of *MdLAR1*, as this gene is known to participate in procyanidin biosynthesis.

## Competing interests

The authors declare that they have no competing interests.

## Authors’ contributions

SAK did the experimental work and have written down the manuscript. JGS and JB helped in the q RT-PCR studies. AA provided the primers for structural genes of the phenylpropanoid pathway. YT made the gene network. EJ provided useful ideas during experiment and the writing process. HJS initiated together with AGB this research, coordinated the project and helped in the writing of the article. All authors read and approved the final manuscript.

## Supplementary Material

Additional file 1 **Genotypes used for measuring relative expression of phenylpropanoid and flavonoid pathway genes and the candidate genes in the mQTL hotspot.** Average sizes of eight fruits per tree genotype are also given at each stage. Click here for file

Additional file 2 **Genotypes used for measuring relative gene expression.** ‘Group A’ had low and ‘Group B’ had high content of ‘Procyanidin dimer II’. The content is given as ^10^log transformed values of ions count per second. Click here for file

Additional file 3 **Corretions betwee the expression levels of genes and metabolite contents in flesh.** The different groups of genes have different colours. Grey: structural genes of the phenylpropaniod pathway. Blue: the putative transcription factor genes that were detected in the mQTL hotspot; Yellow: the putative transcription factor genes that were outside the mQTL hotspot but on LG16; Pink: putative transcription factor genes of the phenylpropanoid pathway that are not located on LG16 but are at other regions of the genome. The metabolites are highlighted in purple and their correlations with the expression levels of all are given in bold text. The metabolites contents (ions count per second) and expression data (expression relatively to the reference gene *MdGAPDH)* were ^10^log transformed before calculation of the correlation coefficients. Scatter plots (data not shown) were made to evaluate the distributions and to exclude outliers that might strongly affect the correlations. the correlations between the expression level of *MdLAR1* and metabolite content are given in bold italics text. Light green colors shows other correlations (r > 0.7) between the expression levels of different genes and also between the expression of different genes and metabolites content. For each metabolite group that had a mQTL at the LG14 hotspot, a representative metabolite is included in this correlation matrix. Click here for file
